# Sequential Navigated Multilocus Repetitive Transcranial Magnetic Stimulation for Concurrent Somatic and Psychiatric Conditions

**DOI:** 10.1097/YCT.0000000000001141

**Published:** 2026-09-10

**Authors:** Satu K. Jääskeläinen, Mi Tram, Tero Taiminen

**Affiliations:** * Division of Medical Imaging, Department of Clinical Neurophysiology, Turku University Hospital and University of Turku; † Department of Psychiatric Neuromodulation, Division of Psychiatry, Turku University Hospital, Turku, Finland

**Keywords:** repetitive transcranial magnetic stimulation, multilocus stimulation, major depression, pain, tinnitus

## Abstract

**Objective:**

Repetitive transcranial magnetic stimulation (rTMS) is efficient in frequently coexisting neurological and psychiatric disorders. This retrospective study investigated potentially additive efficacy and feasibility of multilocus rTMS in patients treated for more than 1 concurrent disorder.

**Methods:**

Thirty-three consecutive patients underwent therapeutic rTMS with several cortical targets for more than 1 disorder. Two patients were excluded (insufficient data). The patients (19 women and 12 men, median age 49 years, range 17–75 years) had combinations of chronic pain (n = 15), depression (n = 30), tinnitus (n = 7), anxiety (n = 6), obsessive-compulsive disorder (n = 3), and eating disorder (n = 3). The combination of pain and depression was most frequent. E-field navigated TMS device (Nexstim Ltd, Helsinki, Finland) was used for a 10-day rTMS-trial (9 in 2 cases). Protocol and cortical target combinations varied according to indications. In addition to clinical evaluation, at least 30% decrease in severity on disorder-specific scales was response limit. All assessments were done at baseline and after 10-day treatment.

**Results:**

Response rate to multilocus rTMS in at least 1 condition was 84%. A response to treatment was observed for both indications in 19 patients, for a single indication in 7 patients, and for none in 5 patients. As available (n = 20), Global Impression of Change was positive in 85% of patients, and 15% reported no change.

**Conclusions:**

Multilocus rTMS is an efficient tool for comorbid neurological and psychiatric disorders, with no serious adverse effects. Responder rate was rather high in patients with comorbidities, suggesting that rTMS efficacy may be associated with inherent patient-related factors.

Repetitive transcranial magnetic stimulation (rTMS) given to disease-specific cortical brain targets is an effective second-line treatment for several treatment-resistant neurological and psychiatric disorders, such as major depression and neuropathic pain.^1^,^2^,^3^ The Canadian Network for Mood and Anxiety Treatments even recommended rTMS as the first-line choice of treatment after failure with 1 drug for major depression.^4^ Recent meta-analyses show that rTMS also improves stroke rehabilitation at the subacute phase and spasticity as well as movement disorders^3^ and treatment-resistant tinnitus.^5^ It may have an effect on several additional psychiatric conditions as well, such as generalized anxiety disorder (GAD),^6^ obsessive-compulsive disorder (OCD),^7^ substance dependence and eating disorders,^3^ and cognitive and negative symptoms of schizophrenia.^3^

Somatic and psychiatric conditions often coexist in various combinations. Similar, overlapping pathophysiologic brain mechanisms, leading to inherent vulnerability to certain neurological and psychiatric disorders, may underlie these conditions.^3^,^8^,^9^,^10^,^11^ Genetic factors influence the efficacy of neuromodulation therapy.^12^ Val66met polymorphism of brain-derived neurotrophic factor (*BDNF*) gene is associated with motor cortex plasticity in acute stroke,^13^ and its Val/Val genotype favors rTMS efficacy in stroke rehabilitation,^14^ whereas Met allele carriers do not show the normal after effects of theta burst stimulation.^15^ In addition, Val/Val genotype enhances neural plasticity and relates to LTP and LTD induction and changes in synaptic efficacy.^12^,^13^ Furthermore, dopamine D_2_ receptor gene (*DRD2*)–related genetic constitution (SNP 957C > T) regulates striatal dopamine level and may also predict treatment response to rTMS.^16^ Homozygous TT genotype of the *DRD2* gene is associated with low thermal pain detection thresholds, increases the risk of neuropathic pain, and is associated with better analgesic response to high-frequency (HF) rTMS targeting the primary somatosensory (S1)/primary motor (M1) cortex.^16^

In clinical practice, rTMS may easily be delivered to several cortical targets during one treatment session, enabling cost-saving treatment of more than one treatment-resistant disorder at the same time.^11^ However, there are scarce data on multilocus rTMS for comorbid disorders during the same therapeutic session. Most studies so far have evaluated the efficacy of single-site rTMS treatment on possible comorbid disorders, for example, anxiety in depression,^17^ depression in pain patients,^11^,^18^ insomnia in GAD,^19^ and major depression in Parkinson disease.^3^,^20^ In many of these studies, single-site rTMS seems to be beneficial for accompanying comorbid symptoms, but the results are not consistent.^21^ This may result from not specifically targeting the optimal brain network for each disorder separately. A few studies have evaluated the efficacy of sequential, multilocus rTMS on affective components of tinnitus (superior temporal gyrus [STG] on the left and the right dorsolateral prefrontal cortex [DLPFC]) with inconsistent results.^22^,^23^,^24^ One small case series (n = 7) on OCD and major depression utilizing sequential targeting of both the right DLPFC and supplementary motor areas (SMAs) bilaterally reported robust therapeutic response in both indications.^25^

We have utilized sequential multilocus rTMS for different combinations of comorbid somatic and psychiatric diseases (pain, major depression, tinnitus, GAD, OCD, and eating disorders) in clinical practice since 2012. Close collaboration between departments of psychiatry and clinical neurophysiology both applying therapeutic rTMS, and cooperation in the multidisciplinary neuromodulation unit of our hospital have given us access to clinical patients from various specialties with comorbid disorder combinations not responding to standard treatments. As rTMS is possibly helpful in many of these indications, multilocus protocols were included in our clinical routine to individually address the needs of each patient.

The aims of this open retrospective clinical study were to evaluate subjective benefits, clinical feasibility, adverse effects, and potentially additive efficacy of sequential multilocus rTMS in psychiatric and somatic patients treated simultaneously for more than 1 indication in naturalistic, real-life settings, without the strict exclusion criteria of randomized controlled trials (RCTs). As many neurologic and psychiatric conditions often occur concurrently in the same patients,^8^ and several of these conditions also seem to respond to therapeutic neuromodulation,^3^ we have conducted a clinical pilot trial with rTMS for multiple indications in various combinations, to investigate whether added clinical benefit could be achieved with multilocus rTMS treatment. Figures 1 and 2 show the diagnostic combinations and cortical targets applied in these patients, and Table 1 summarizes the treatment protocols utilized in the therapeutic sequential multilocus rTMS for depression, chronic pain, tinnitus, GAD, OCD, and eating disorders, present in different combinations in the patients evaluated in this study. Additionally, we wanted to test a hypothesis that rTMS treatment response is determined by inherent constitutional, possibly genetic within-subject factors, which would be reflected in a finding that, regardless of the different diagnostic combinations, in the majority of cases, the patients would either benefit from rTMS treatment in all concurrent treatment indications or not at all.

**FIGURE 1:**
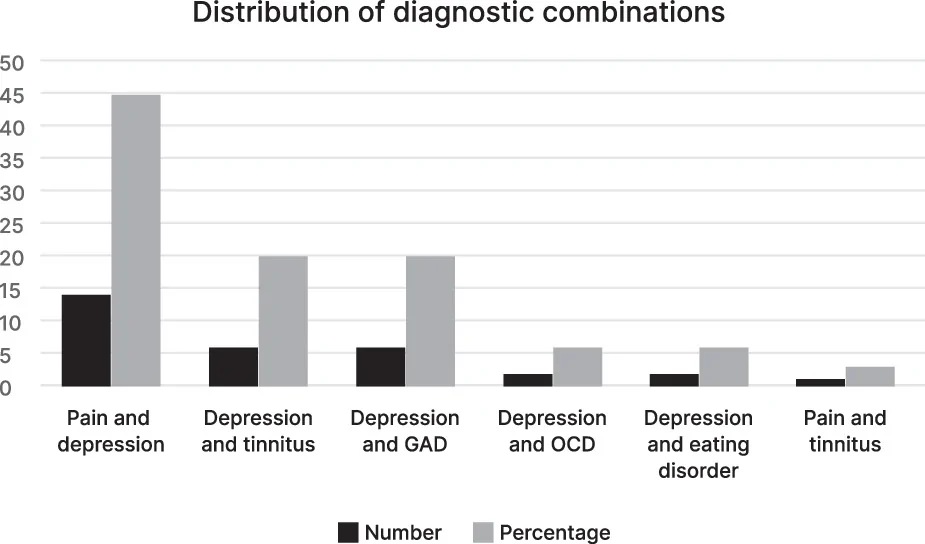
Distribution of the main diagnostic combinations of comorbid somatic or psychiatric disorders (n = 31).

**FIGURE 2:**
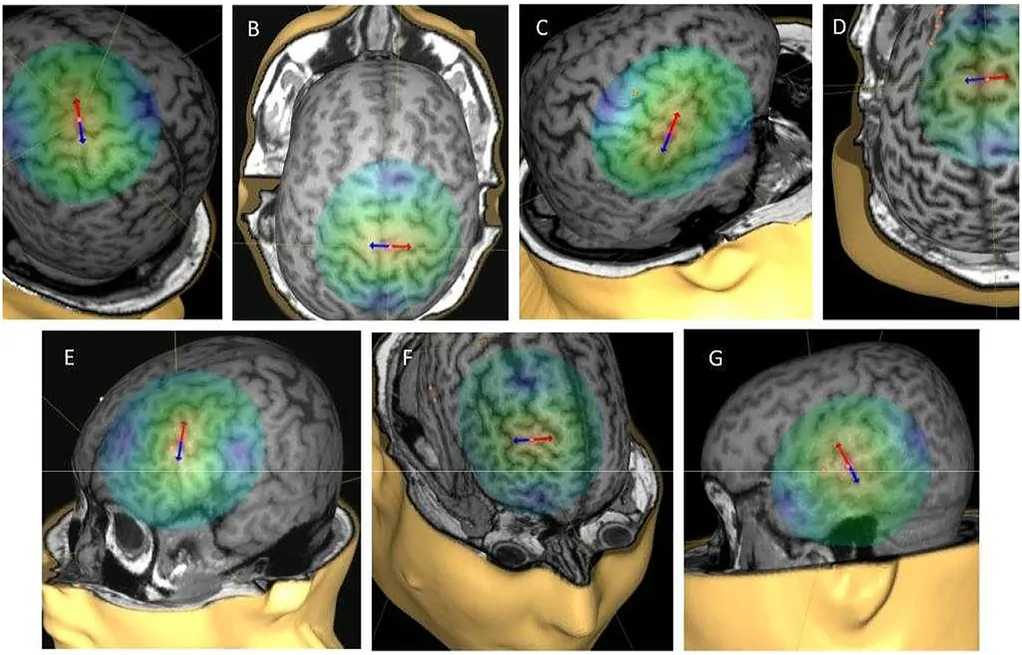
Cortical targets of navigated multilocus rTMS for the different treatment indications. A = motor cortex (M1) hotspot for treating pain in the right hand; the same spot on the right M1 was used when the pain was in the left hand, B = M1 for pain in the left lower limb; the same spot on the left M1 and direction of the E-field toward the left ear was used to treat pain in the right lower limb, C = right operculoinsular cortex (“S2”) for pain; this target was applied, if M1 treatment had not been effective during the first 5 days, D = pre-SMA for OCD, E = left DLPFC for major depression (HF-rTMS), F = right DLPFC for GAD or depression with anxiety (LF-rTMS), G = left superior temporal gyrus for tinnitus, the more posterior target is for high-pitched tinnitus. Red arrowhead indicates the site and direction of the TMS-induced E-field vector that was always at right angles against the underlying gyral line.

**TABLE 1:** Protocols for Individually Tailored rTMS Treatment

Indication	Primary Target	Optional Secondary Target	Frequency	Intensity (% of RMT)	No. of Pulses	No. of Sessions
Pain	Contralateral M1 (hand/foot/face representation area)	“S2” right parietal operculum Left DLPFC*	10 Hz	75%–90%	1500–3000	10–15
Depression	Left DLPFC (BA 46/9)	Right DLPFC (BA 46/9)	Left 10 Hz Right 1 Hz	75%–110%	1000–5000	15–25
Anxiety (GAD)	Right DLPFC (BA 46/9)		1 Hz	90%–110%	1200–3000	15–25^†^
Tinnitus	Left STG (auditory cortex)	Right STG	1 Hz	85%–100%	1000–3000	10–15
OCD	Bilateral pre-SMA		1 Hz		1500/side	15^†^
ED	Left DLPFC (BA 46/9)		10 Hz	80%–100%	2000–3000	10

ED, eating disorder (2 anorexia, 1 bulimia). Intensity adjusted according to tolerance to discomfort caused by the stimulation. Number of pulses for each target during 1 session; total number of pulses during 1 session did not exceed 6000. The stimulation trains for each target were separated with a 10-minute break halfway the rTMS series, during which the other target was treated. *In 1 fibromyalgia patient and in 1 migraine patient. ^†^Only 9 sessions in 1 patient.

## METHODS

### Patient Characteristics

This retrospective clinical evaluation is based on patient records of 33 consecutive patients treated with navigated multilocus rTMS for more than 1 indication during the years 2012–2017 at the departments of clinical neurophysiology (CN) and psychiatry of Turku University Hospital. Inclusion criteria were as follows: (1) patient had received an intensive rTMS treatment series (daily sessions for 9–10 days) for at least 2 different indications, psychiatric and/or somatic, and with more than 1 cortical target, given at the same treatment session, and (2) there were enough data on the patient records to assess treatment response for the main indications at the end of the intensive treatment period on day 10 (day 9 in 2 patients). For 2 patients, there were not enough data for the analysis of combination treatment effects. One of them, a 49-year-old woman with major depression and pain (complex regional pain syndrome of the right hand), received only treatment for pain, because she could not tolerate stimulation of the DLPFC targets after trials on the left and right side during the first few sessions. She did not benefit from rTMS for pain. For another patient (a 51-year-old man) with major depression and anxiety, there were not enough data in the patient files to analyze response to GAD, but he did not benefit from rTMS to major depression and reported no subjective benefits. Thus, these 2 excluded patients did not benefit from rTMS in any indication.

The final sample size in the statistical analyses was 31 patients with enough follow-up data, 19 women and 12 men, with an age range from 17 to 75 years (median, 49 years). The diagnostic combinations of the patients, grouped by the 2 main treatment indications, are shown in Figure 1. All patients underwent a 3-dimensional head magnetic resonance imaging (MRI) to provide anatomical guidance for navigated rTMS. There were no pathological structural findings in the MRI scans except for the 1 patient with multiple sclerosis (MS). All patients gave their informed consent for rTMS in other indications than major depression; after 2014, informed consent was not asked for neuropathic pain.

### Assessment of Symptoms and Treatment Effects

The symptom severity was assessed at baseline and after 5 and 10 days of rTMS treatment (9 days in 2 patients). In all analyses, of both the clinical impression and the results in the assessments with numerical scales, the results gathered immediately at the end of the induction phase were used. Table 2 summarizes the assessment tools and their interpretation.

**TABLE 2:** Summary of the Symptom Assessment Tools and Their Scoring

Symptom	Assessment Tool	Response Limit
Pain intensity	VAS or NRS	30% decrease from baseline
Pain interference	VAS or NRS	30% decrease from baseline
Tinnitus intensity	VAS or NRS	30% decrease from baseline
Tinnitus interference	VAS or NRS	30% decrease from baseline
Depression	BDI	30% decrease from baseline
Depression	MADRS	30% decrease from baseline
Generalized anxiety	BAI	30% decrease from baseline
Obsessive-compulsive disorder	Y-BOCS	20% decrease from baseline
Patients' Impression of Change	GIC	At least +1
Eating disorders (anorexia, bulimia)	Clinical assessment: changes in weight and eating habits	Doctor's impression of positive change

GIC compared with baseline (7-step categorical scale: −3 very much worse, 0 = no change, +3 very much improved compared with baseline). At least 30% decrease in symptom severity is an often applied cut-off point in pain treatment studies. It was also used to psychiatric scores because the 10-day induction phase, that is, the Evaluation period used here, was shorter than normally adopted in psychiatric rTMS trials. BAI, Beck Anxiety Inventory; BDI, Beck Depression Inventory (0–30); MADRS, Montgomery-Åsberg Depression Rating Scale; NRS, numerical rating scale (0- to 11-step, categorical); VAS, visual analog scale (0–100 mm, linear).

The intensity and interference caused by pain and tinnitus were assessed with visual analog scale from 0 to 100 mm, with 0 indicating no pain/tinnitus sound, and 100 indicating the worst imaginable pain/tinnitus sound. In addition, numerical rating scales were applied in patient diaries, with an 11-step rating of symptoms from 0 (none) to 10 (most severe imaginable). Depression was assessed with Beck Depression Inventory and Montgomery-Åsberg Depression Rating Scale. GAD and OCD were scored with Beck Anxiety Inventory and Yale-Brown Obsessive Compulsive Scale (Y-BOCS). The 7-step patient's Global Impression of Change (GIC; −3 = very much worse, 0 = no change, +3 = very much better than before treatment) score was available for patients with pain and tinnitus. Patient records were additionally utilized to register and analyze subjective benefit in psychiatric indications, especially OCD and eating disorders (amount of obsessive behaviors and thoughts, weight, eating habits) immediately at the end of the induction phase. All centrally acting medications were registered.

A response was defined as at least 30% decrease in disorder-specific symptom scores at the end of the rTMS induction phase, except for Y-BOCS, in which a 20% decrease was considered a response, because OCD patients are typically very treatment resistant. Complete numerical data for all concurrent therapeutic indications, at the baseline and the end of the induction phase, were available for 23 patients, for whom comprehensive analyses with percentage changes could be done. Due to open, retrospective study design, complete numerical data were available only for the patients treated at the department of CN, but less consistently for patients treated at the department of psychiatry. However, we could determine an outcome score: responder or not to each treatment indication, for all 31 patients at the end of the induction phase of 10 (9) days. This final overall score utilized, in addition to all available numerical ratings, the patient files with doctor's impression of change combined with patient's own subjective experience of beneficial effects on daily life, mood, anxiety, behaviors, motor abilities, weight, eating habits, and sleep. The final score allowed us to classify whether the patient had benefitted from the treatment in 1, 2, or none of the indications after 2 weeks of therapeutic rTMS.

### Navigated rTMS

A commercially available E-field navigated NBS System 4.0 (Nexstim Ltd, Helsinki, Finland; CE Mark in the EU for pain and depression therapy) was used for rTMS treatment. With this device, the user can control the cortical target and the direction of the induced electric current vector online during stimulation. With this device, the “hotspot” of stimulation has an anatomical accuracy of a few millimeters.^26^ During the first session, a physician determined the resting motor threshold (RMT) at the right M1 cortex representing the left hand thenar muscles using a figure-of-8 coil giving biphasic magnetic pulses. The RMT was defined as the lowest intensity (% of maximum device output) capable of eliciting >50 μV motor evoked potential in 50% of the trials. Details of the RMT determination have been previously described.^27^,^28^,^29^

At least 10 treatment sessions over 2 weeks (5 daily weekday sessions) were given to 29 patients, and 2 patients received 9 treatment sessions during the induction phase. The sequential multilocus rTMS was as an add-on therapy, and possible other treatments did not change during the intensive treatment phase. Trained CN technicians and psychiatric nurses gave serial treatments.

The treatments were given to several cortical targets shown in Figure 2 and listed in Table 1 with either low-frequency (LF) rTMS at 1 Hz or HF rTMS at 10 Hz. Targets and frequencies were tailored individually according to the indications. Table 1 describes in detail the therapeutic rTMS protocols used at each cortical target for different indications: pain, major depression, tinnitus, GAD, OCD, and eating disorders. In addition to the cortical targets applied in the study, Figure 2 shows, at each target location, the position and direction of the induced electric current field vectors that were always at right angles against the corresponding gyral line.

Pain treatment was always started with HF rTMS to the contralateral primary motor cortex (M1) representing the main pain area (face, hand, trunk, foot). If there was no response or the pain got worse during the first week, the stimulation target was changed to the right parietal opercular cortex overlying the S2 cortex and posterior insula, “S2.”^27^,^30^ For pain, the stimulation intensity was always below RMT, in most cases 90% RMT at the M1 and 80% to 85% at the right “S2.” Intensity was lowered if the patient could not tolerate higher intensities because of pain or disturbing facial and masticatory muscle activation.

For major depression, the left DLPFC (border of Brodmann areas [BA] 46/9) was the primary target of HF rTMS according to the guidelines by Mylius et al.^31^ If the patient had GAD or disturbing anxiety with major depression, LF rTMS to the right DLPFC (BA 46/9) was included in the protocol. On the left side, the intensity was 90% to 110% of the RMT in most patients, but in some patients (with high RMT), it had to be kept lower because of pain or disturbing muscle contractions. On the right side, the intensity was generally higher, 100% to 110% RMT.

For tinnitus, the left STG was the primary target for LF rTMS, but in 2 patients, the right STG was added to the protocol because the treatment lateralized the tinnitus. The intensity at the STG was in most patients between 90% and 100% RMT.

For OCD, bilateral LF rTMS stimulation at the pre-SMA was used, but the exact intensity of the treatment was not registered (3 patients). The target site for eating disorders was the left DLPFC with HF rTMS applied at an intensity between 80% and 100% RMT.

At the beginning of the observation time (years 2012–2016), pulse numbers were higher and given continuously to each target at a time. Since August 2016, a 10-minute break was introduced to the rTMS series at each target site implying 2 repeated stimulations with potentially better efficacy. During the break, the other treatment target was stimulated. Since 2017, the number of pulses/target was limited to 1500, and the total number of pulses/session to 4500.^32^

### Statistical Analyses

Statistical analyses were performed for the 31 patients with at least 9 treatment sessions for at least 2 indications and for whom appropriate data were available from the symptom scores and patient files. Effects of age of the patients on treatment efficacy was analyzed by comparing groups of responders and nonresponders with Student *t* test in the whole group (n = 31) and, additionally, with mixed procedure analysis of variance for the group with complete numerical data (n = 23). Effect of sex on treatment response was investigated with *χ* ^2^ test (n = 31), and Fisher exact test and mixed-procedure analysis of variance taking into account both the age and sex of the subjects (n = 23). Correlation of percentage alterations in symptom scores for the 2 main treatment indications was evaluated with Pearson or Spearman correlation analysis in the subgroup of patients with complete numerical data available. SAS system software (SAS Institute Inc, Cary, NC) was used in the data analyses.

## RESULTS

### Patient Characteristics and Multilocus rTMS Treatment Protocols

In the whole group of 33 patients, for 2 patients there were not enough data for the analysis of combination treatment effects. One of them, a 49-year-old woman with major depression and pain (complex regional pain syndrome of the right hand), received only treatment for pain, because she could not tolerate stimulation of the DLPFC targets after trials on the left and right side during the first few sessions. She did not benefit from rTMS for pain. For another patient (a 51-year-old man) with major depression and anxiety, there were not enough data in the patient files to analyze response to GAD, but he did not benefit from rTMS to major depression and reported no subjective benefits. Thus, these 2 excluded patients did not benefit from rTMS in any indication. The final sample size in the analyses was 31 patients with enough follow-up data and at least 9 days of intensive rTMS for more than 1 indication. Table 3 shows the demographic data of these patients with medications.

**TABLE 3:** Demographic Data of the Patients, Their Clinical Diagnoses, and Medications With Corresponding Indications

Sex	19 Women, 12 men	
Age	Median 49 (17–75) y	
Duration of the disorder	Median 11 (1–40) y	
Diagnoses	Chronic pain	15 Patients
	Major depression	30 Patients
	GAD	7 Patients
	OCD	3 Patients
	Tinnitus	8 Patients
	Eating disorder	3 Patients (1 bulimia, 2 anorexia)
Medications:		Indications:
Opioids	10/31 (6 weak, 5 strong)	Pain
A2δ ligands	7/31	Pain, GAD (1), OCD (1)
Other antiepileptic drugs	4/31	Depression
Benzodiazepines	17/31	All diagnostic categories
SSRI/SNRI	10/31	Depression, anorexia (1), OCD (1)
Antipsychotic drugs	14/31	All diagnostic categories

A2δ ligands, pregabalin or gabapentin; SSRI/SNRI, selective serotonin reuptake inhibitors/serotonin and noradrenaline reuptake inhibitors.

There were 6 different combinations of diagnoses planned to be treated in the patients analyzed (Fig. 1). The 3 larger subgroups were (1) pain and major depression (n = 14, 4 of these patients also had GAD, and 1 had tinnitus), (2) major depression and GAD (n = 6, one of them also had OCD, and 1 had bulimia), and (3) tinnitus and major depression (n = 6). In addition, there were 2 patients with OCD and major depression, 2 patients with anorexia nervosa and major depression (one of them also had dissociative disorder), and 1 patient with central pain, spasticity, and tinnitus due to MS. Pain diagnosis was most often definite neuropathic pain (n = 7/14, diagnosis confirmed with electroneuromyography). Additionally, there were 3 patients with fibromyalgia and 4 patients with other, chronic widespread pain conditions.

### Response to Navigated Multilocus rTMS Treatment

Table 4 summarizes the responses to therapeutic rTMS in different diagnostic combinations. In the total sample of 31 patients, 26 patients (84%) benefitted in at least 1 indication. Of these 26 patients, 19 benefitted in all indications, and 7 in only 1 indication. Five patients did not respond to navigated multilocus rTMS. Thus, the majority of 26 responders (19/26, 73%) benefitted in more than 1 indication. On the other hand, from the total sample of 31 patients, 77% either benefitted in all indications treated or not at all. In the whole group (n = 31), the type of response (response or no response) to rTMS did not depend on the age (*P* = 0.1601) or sex of the patients (*P* = 0.5358). These effects were further analyzed in more detail in those patients with complete numerical data available (below).

**TABLE 4:** Summary of the Results in Different Combinations of Comorbid Disorders (Numbers in Parentheses Refer to Patient Number, Total Number of Patients Was 31)

Diagnostic Combination of Disorders	Therapeutic Efficacy of Multilocus rTMS
Pain and depression (8)	5/8 Response to both 3/8 Response to one
Tinnitus and depression (6)	4/6 Response to both 2/6 Response to one
Depression and GAD (5*)	2/5 Response to both 3/5 No response
Pain, depression and GAD (5)	5/5 Response to at least 2 indications
Depression and OCD (2)	1/2 Response to both 1/2 No response
Eating disorder (3; anorexia: 1 with depression, and 1 with depression and dissociative disorder; bulimia with depression and GAD with OCD traits)	1/3 Response to all (bulimia, depression, OCD) 1/3 Response to 1 (dissociative disorder) 1/3 No response (anorexia, depression)
Pain and tinnitus (1^†^)	1/1 Response to both (also spasticity improved)
Pain, depression, and tinnitus (1^‡^)	1/1 Response to 1 (tinnitus improved)
Altogether (31)	19/31 Response to both main indications 7/31 Response to 1 indication 5/31 No response to any indication

*One of these patients also had OCD that did not improve. ^†^Symptoms due to MS. ^‡^Multiple symptoms due to traumatic brain injury.

### Responder Rates According to Diagnostic Combinations

As regards the responder rates in different diagnostic combinations, the subgroups with major depression and pain (n = 14), major depression and tinnitus (n = 6), or major depression and GAD (n = 6) seemed to respond best to therapeutic neuromodulation, as shown in Table 4. The majority of the patients in these combination treatments responded to both treatment indications (62%), and the remaining ones mostly to 1 indication (31%). There were 3 nonresponders in the subgroup with depression and GAD (one of them severely suicidal).

Combination treatment for the few patients with eating disorders gave more variable results. Only the patient with bulimia, major depression, GAD, and OCD responded to rTMS treatment in all indications. The 2 anorexia nervosa patients did not benefit in any of their treatment indications (weight, GAD, major depression, and dissociative disorder). Of the 3 OCD patients, one did not benefit at all, whereas 2 showed benefit in OCD symptoms, and one of them also had a significant decrease in GAD symptoms. Good response to multilocus rTMS was not restricted to patients with psychiatric comorbidities; 1 patient had only somatic disorders with excellent response to rTMS, his central pain and spasticity vanished, tinnitus intensity decreased by 59%, and GIC was +2.

### Patients With Complete Numerical Follow-up Data

**FIGURE 3:**
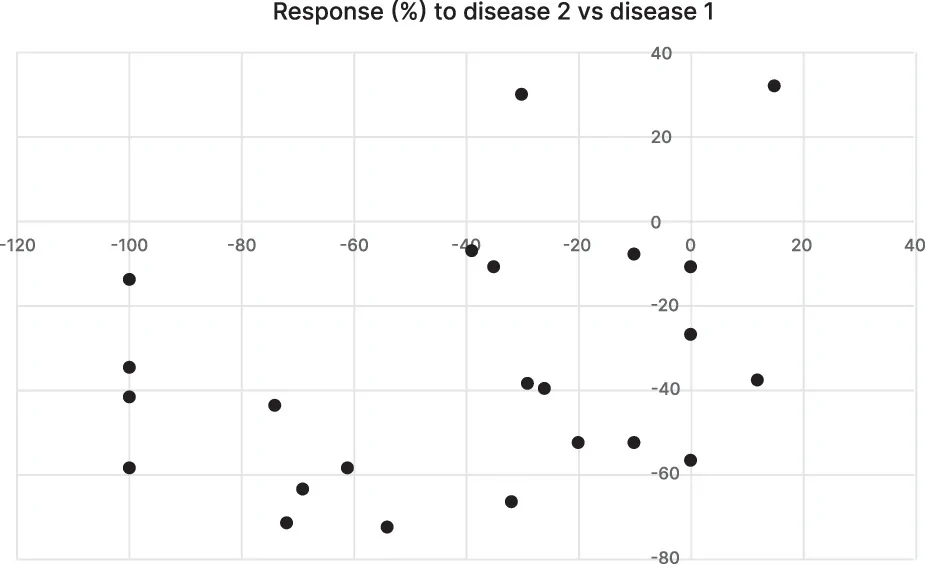
Relationship of responses to rTMS given for 2 different concurrent disorders. Results at the end of the intensive induction phase of rTMS for 2 separate treatment indications, reflected as percentage changes from baseline in 23 patients with complete numerical follow-up data. *Y*- and *X*-axis represent the % change in symptoms for the 2 disorders treated; negative % values indicate that the treatment resulted in a decrease in the disease-specific symptom scores during the 10-day rTMS period. There was only 1 patient who did not benefit at all and showed increase in both symptom scores (in the upper right corner of the figure).

Of the 23 patients with complete numerical data available for all therapeutic indications, 3 did not respond in any of the indications, in 8 patients, rTMS was effective in 1 indication, and 12 patients responded in at least 2 indications. Numerical percentage decreases in the symptom scores for the 2 main therapeutic indications showed a vague positive association (Fig. 3) that remained nonsignificant in Spearman correlation analysis (*r* = 0.35, *P* = 0.1028). The response profile, response to 1, 2, or none of the indications, was not associated with sex (Fisher exact test *P* = 0.8304). However, the percentage decrease in symptom severity depended on sex, as the estimate of mean response reduction was 67.8% (SE, 11.5%) for indication 1 in women, and only 26.1% (SE, 8.4%) in men (mixed procedure, *P* = 0.0090). For indication 2, sex difference was to the same direction but not significant (*P* = 0.1619). Age did not seem to be associated with response rate in any of the indications (Pearson *r* = −0.26/−0.25, with *P* = 0.2266/0.2502). However, when analyzed against response profile, age had a borderline effect. The patients with no response to any indication were younger (estimate of mean, 35.3 [SE, 7.98] years) than those who benefitted in both indications (mean, 56.7 [SE, 3.99] years) (mixed procedure fixed effects *P* = 0.0477, Tukey-Kramer adjusted *P* = 0.0661). This statistical association of better efficacy to older age may depend on the more difficult treatment indications in the younger patients, including all OCD and eating disorder patients. The effects of diagnoses were not analyzed statistically because of small number of patients.

### Adverse Effects

There were no major adverse effects in the 31 patients; 19 patients did not report any side effects. The single complaint in 8 patients was tension-type headache or migraine, and 2 patients described tiredness and nonspecific irritation/paresthesia. Two patients felt changes in tinnitus sound: lateralization to the ear opposite to the treatment side and high-pitched interfering peaks in the former monotonous tinnitus sound that decreased in intensity. The 2 shorter 9-day treatments were due to logistic troubles; one of these patients found frequent visits to the hospital uncomfortable.

## DISCUSSION

### Multilocus rTMS—A Feasible and Effective Therapy for Comorbid Disorders?

Overall, this retrospective clinical study shows that sequential multilocus rTMS is suitable and feasible for simultaneous treatment of multiple concurrent somatic and psychiatric disorders during the same treatment session. Furthermore, response to 1 indication may predict overall favorable response to the other indications as well. On the other hand, poor response to 1 indication seemed to be associated with treatment failure also in the other indications. So, rTMS efficacy may be determined by within-patient factors, possibly genetic,^13^,^14^,^15^,^16^ and related to individual tendency for brain level neural plasticity, as the responder patterns were not clearly associated with the diagnostic combination under treatment, age, or sex of the patients. Nevertheless, in line with some previous reports, also the present results on a very small number of patients suggest that eating-related symptoms of anorexia nervosa may be difficult to treat also with rTMS, although comorbid depression or anxiety may benefit from the treatment.^33^,^34^

Although there is a known, high comorbidity of several disorders that have been shown to benefit from therapeutic rTMS, to our knowledge, there are very scarce data on the potential benefits of sequential multilocus rTMS targeting different cortical sites at the same session for comorbid disorders. Some studies have shown trends, not always reaching statistical significance, toward better outcomes with sequential multisite stimulation additionally for affective components of tinnitus patients,^22^,^23^,^24^ or OCD with comorbid major depression,^25^ but findings remain inconclusive, partly because of small number of patients.

A few articles and meta-analyses on single-target rTMS protocols have reported mostly beneficial alterations in accompanying comorbid symptoms, for example, depression or anxiety in chronic pain patients,^11^,^18^ anxiety or OCD in patients with major depression,^17^,^35^ or major depression in patients with Parkinson disease.^20^ However, in all these studies, only 1 brain site for the main treatment indication has been stimulated, not the optimal targets for each comorbid disorder separately, which might add efficacy. Even when taking into account the open-study design, the exceptionally good percentage of responders in the present material suggests that the use of more than 1 cortical target may improve the efficacy of rTMS in patients with comorbid disorders. Specially, combining HF rTMS to M1 or “S2” pain targets with left HF DLPFC in patients with chronic pain and depression, left HF DLPFC and right LF DLPFC targets in patients with depression and GAD, and left LF STG and left HF DLPFC in patients with tinnitus and depression resulted in clinically meaningful reduction of symptom scores for the treatment indications in the majority of these patients.

### Comorbidity—Similar Brain-Level Mechanisms Behind Different Disorders

Interestingly, our results imply that therapeutic neuromodulation may be especially efficient in patients with comorbid diseases that according to functional imaging, neurotransmitter and electroencephalogram studies share many overlapping pathophysiological central nervous system mechanisms. Shared vulnerability to coexisting conditions due to similar alterations in neurotransmitter systems and neural plasticity, connectivity, or resting state networks of the brain have been suggested to underlie comorbid depression and pain,^8^,^11^ tinnitus and pain,^9^,^36^ or depression and GAD,^17^,^35^ or OCD.^25^,^35^ It could be reasoned that additive efficacy may be achieved while targeting rTMS at several hubs of the disturbed brain networks, which could explain to some extent the exceptionally high response rate in the present study (84%). The capacity to neuroplasticity of the brain may, in maladaptive conditions, lead to comorbid chronic disorders but has also been shown crucial in mediating the therapeutic benefits of therapeutic neuromodulation utilizing neuroplasticity^12^,^13^,^14^ to restore lost balance and network connectivity in the brain, and boosting top-down control via endogenous neurotransmitter systems.^16^ In addition to the diagnostic combinations described here, recent studies show interesting possibilities for future application of more specific and potentially more efficient multilocus rTMS approach to treat, for example, insomnia in GAD patients,^19^ cognitive deficits in neuropsychiatric conditions,^37^ or depression in dementia.^38^

### Navigated rTMS May Improve Therapeutic Efficacy in Comorbid Disorders

We used individual 3-dimensional head MRI for neuronavigated rTMS that is associated with better response rates in patients with neuropathic pain^39^ and major depression.^40^ This may also have influenced the high incidence of responders, despite the short 10 (9)-day treatment period—normally, much longer series (25 to >30 sessions) are used for depression, OCD, and GAD. A period of 10 days was chosen, as these data were available for most of the patients who had undergone sequential multilocus rTMS in our unit. Longer treatment attempts might have given better results in some of the patients who now remained slightly below the preset responder limits. In fact, the majority of the patients with response to 1 indication who continued to maintenance therapy showed later responsiveness also to the other treatment indications (unpublished observation).

The responder rate in the present study on comorbid disorders is higher (84%) than the responder rates generally reported in open-label rTMS studies (61%).^41^ This suggests potential bidirectional, additive efficacy of multilocus rTMS, covering both biological and psychological dimensions of the disorders and boosting resilience reciprocally on both axes. In addition, naturalistic clinical setting enables a more realistic assessment of the potential utility of individually tailored multilocus rTMS for comorbid disorders than refined RCT designs with strict exclusion criteria.

At closer look of those patients with complete numerical follow-up data available, female patients seemed to show more pronounced decrease in symptom-specific scores than male, in line with a meta-analysis showing that, in major depression, better efficacy of rTMS is associated with female sex.^42^ However, the sex difference in a subset of this small material cannot be regarded more than suggestive, but it could be associated with the fact that most of the diagnostic combinations included major depression. However, excellent response to both indications also occurred in a male patient with pain and comorbid tinnitus.

### Strengths and Limitations of the Study

There are several limitations to this study with uncontrolled, retrospective, and open design; heterogeneity of the clinical conditions treated; and individually adjusted rTMS procedures applied. In addition, the evaluation of treatment effects in all therapeutic indications was precise in only 23 patients with complete numerical scores available at the end of the induction phase (all treated at the department of clinical neurophysiology). In the remaining patients, the response to treatment was less consistently assessed with numerical scales and relied partly on overall clinical evaluation and patients' impression of change (at the department of psychiatry). Furthermore, for the whole group, only short-term effects at the end of the 10-day induction phase were available to assess the efficacy of the treatment.

On the other hand, this material elucidates the clinical value of therapeutic rTMS in a challenging patient population with several concurrent treatment-resistant neurological and psychiatric disorders, in real-life naturalistic settings. In the clinical routine of our neuromodulation units, it has been feasible and easy to use individually adjusted protocols utilizing add-on multilocus rTMS, targeting the optimal brain locations with neuronavigation during the same session for treatment of distinct symptom combinations. These interventions were accomplished without exclusion of somatic patients who also have major psychiatric comorbidities and without medication-based restrictions occurring in the exclusion criteria of RCTs. This approach provided cost-effective application of therapeutic neuromodulation for multiple treatment-resistant conditions during a single rTMS session.

## CONCLUSIONS

Individually tailored sequential multilocus rTMS seems to be clinically feasible, effective, cost-saving, and safe therapeutic tool, with no major adverse effects, for combination treatment of treatment-resistant, often comorbid somatic and psychiatric disorders. However, because of the small number of patients, the present results require further confirmation in prospective clinical studies on larger patient samples. Based on these preliminary data, it seems that comorbid combinations, including major depression with pain, tinnitus, or GAD, might provide appropriate targets for future research on the use of multilocus rTMS. As chronic pain and depression are both very prevalent disorders, and they often coexist, it would also offer a good target to study the cost-efficacy of multilocus rTMS as this method has been suggested to result in greater reduction in direct healthcare costs when used for combined disorders.^11^ The future studies may hopefully establish the full potential of rational and cost-effective,^11^ individually adjusted treatment with accurate neuronavigated multilocus rTMS during the same therapeutic session.

## References

[1] Slotema CW Blom JD Hoek HW . et al, Should we expand the toolbox of psychiatric treatment methods to include repetitive transcranial magnetic stimulation (rTMS)? A meta-analysis of the efficacy of rTMS in psychiatric disorders. J Clin Psychiatry. 2010;71:873–884. 20361902 10.4088/JCP.08m04872gre

[2] Cruccu G Garcia Larrea L Hansson P . EAN guidelines on central neurostimulation therapy in chronic pain conditions. Eur J Neurol. 2016;23:1489–1499. 27511815 10.1111/ene.13103

[3] Lefaucheur JP Aleman A Baeken C . Evidence-based guidelines on the therapeutic use of repetitive transcranial magnetic stimulation (rTMS): an update (2014-2018). Clin Neurophysiol. 2020;131:474–528. 31901449 10.1016/j.clinph.2019.11.002

[4] Milev RV Giacobbe P Kennedy SH . Canadian Network for Mood and Anxiety Treatments (CANMAT) 2016 clinical guidelines for the management of Adults with major depressive disorder: section 4. Neurostimulation treatments. Can J Psychiatry. 2016;61:561–575. 27486154 10.1177/0706743716660033 PMC4994792

[5] Soleimani R Jalali MM Hasandokht T . Therapeutic impact of repetitive transcranial magnetic stimulation (rTMS) on tinnitus: a systematic review and meta-analysis. Eur Arch Otorhinolaryngol. 2016;273:1663–1675. 25968009 10.1007/s00405-015-3642-5

[6] Cui H Jiang L Wei Y . Efficacy and safety of repetitive transcranial magnetic stimulation for generalised anxiety disorder: a meta-analysis. Gen Psychiatr. 2019;32:e100051. doi:10.1136/gpsych-2019-100051. 31673675 PMC6802976

[7] Zhou S Fang Y . Efficacy of non-invasive brain stimulation for refractory obsessive-compulsive disorder: a meta-analysis of randomized controlled trials. Brain Sci. 2022;12:943. doi:10.3390/brainsci12070943. 35884749 PMC9313124

[8] Taiminen T Forssell H Tenovuo O . Psychiatric comorbidity in atypical facial pain and burning mouth syndrome. Scand J Pain. 2011;2:155–160. 29913754 10.1016/j.sjpain.2011.06.004

[9] De Ridder D Elgoyhen AB Romo R . Phantom percepts: tinnitus and pain as persisting aversive memory networks. Proc Natl Acad Sci U S A. 2011;108:8075–8080. doi:10.1073/pnas.1018466108. 21502503 PMC3100980

[10] Sahlsten H Taiminen T Karukivi M . Psychiatric (Axis I) and personality (Axis II) disorders and subjective psychiatric symptoms in chronic tinnitus. Int J Audiol. 2018;57:302–312. 29188734 10.1080/14992027.2017.1409440

[11] Leung A Shirvalkar P Chen R . Transcranial magnetic stimulation for pain, headache, and comorbid depression: INS-NANS expert consensus panel review and recommendation. Neuromodulation. 2020;23:267–290. 32212288 10.1111/ner.13094

[12] Ridding MC Ziemann U . Determinants of the induction of cortical plasticity by non-invasive brain stimulation in healthy subjects. J Physiol. 2010;588:2291–2304. 20478978 10.1113/jphysiol.2010.190314 PMC2915507

[13] Di Lazzaro V Pellegrino G Di Pino G . Val66Met BDNF gene polymorphism influences human motor cortex plasticity in acute stroke. Brain Stimul. 2015;8:92–96. 25241287 10.1016/j.brs.2014.08.006 PMC4813754

[14] Chang WH Bang OY Shin Y-I . BDNF polymorphism and differential rTMS effects on motor recovery of stroke patients. Brain Stimul. 2014;7:553–558. 24767962 10.1016/j.brs.2014.03.008

[15] Cheeran B Talelli P Mori F . A common polymorphism in the brain-derived neurotrophic factor gene (BDNF) modulates human cortical plasticity and the response to rTMS. J Physiol. 2008;586:5717–5725. 18845611 10.1113/jphysiol.2008.159905 PMC2655403

[16] Jääskeläinen SK Lindholm P Valmunen T . Variation in the dopamine D_2_ receptor gene plays a key role in human pain and its modulation by transcranial magnetic stimulation. Pain. 2014;155:2180–2187. 25180011 10.1016/j.pain.2014.08.029

[17] Clarke E Clarke P Gill S . Efficacy of repetitive transcranial magnetic stimulation in the treatment of depression with comorbid anxiety disorders. J Affect Disord. 2019;252:435–439. 31003113 10.1016/j.jad.2019.03.085

[18] Hsu JH Daskalakis ZJ Blumberger DM . An update on repetitive transcranial magnetic stimulation for the treatment of co-morbid pain and depressive symptoms. Curr Pain Headache Rep. 2018;22:51. 29904802 10.1007/s11916-018-0703-7

[19] Huang Z Li Y Bianchi MT . Repetitive transcranial magnetic stimulation of the right parietal cortex for comorbid generalized anxiety disorder and insomnia: a randomized, double-blind, sham-controlled pilot study. Brain Stimul. 2018;11:1103–1109. 29871798 10.1016/j.brs.2018.05.016

[20] Randver R . Repetitive transcranial magnetic stimulation of the dorsolateral prefrontal cortex to alleviate depression and cognitive impairment associated with Parkinson's disease: a review and clinical implications. J Neurol Sci. 2018;393:88–99. 30149227 10.1016/j.jns.2018.08.014

[21] Lindholm P Lamusuo S Taiminen T . The analgesic effect of therapeutic rTMS is not mediated or predicted by comorbid psychiatric or sleep disorders. Medicine. 2016;95:e5231. doi:10.1097/MD.0000000000005231. 27858874 PMC5591122

[22] Kleinjung T Eichhammer P Landgrebe M . Combined temporal and prefrontal transcranial magnetic stimulation for tinnitus treatment: a pilot study. Otolaryngol Head Neck Surg. 2008;138:497–501. 18359361 10.1016/j.otohns.2007.12.022

[23] Lehner A Schecklmann M Poeppl TB . Multisite rTMS for the treatment of chronic tinnitus: stimulation of the cortical tinnitus network—a pilot study. Brain Topogr. 2013;26:501–510. 23229756 10.1007/s10548-012-0268-4

[24] Kreuzer PM Poeppl TB Vielsmeier V . The more the merrier? Preliminary results regarding treatment duration and stimulation frequency of multisite repetitive transcranial magnetic stimulation in chronic tinnitus. Prog Brain Res. 2021;262:287–307. 33931185 10.1016/bs.pbr.2021.01.021

[25] Tadayonnejad R Wilson AC Corlier J . Sequential multi-locus transcranial magnetic stimulation for treatment of obsessive-compulsive disorder with comorbid major depression: a case series. Brain Stimul. 2020;13:1600–1602. 33065361 10.1016/j.brs.2020.10.003

[26] Lefaucheur JP Picht T . The value of preoperative functional cortical mapping using navigated TMS. Neurophysiol Clin. 2016;46:125–133. 27229765 10.1016/j.neucli.2016.05.001

[27] Valmunen T Pertovaara A Virtanen A . Modulation of facial sensitivity by navigated rTMS in healthy subjects. Pain. 2009;142:149–158. 19201092 10.1016/j.pain.2008.12.031

[28] Sahlsten H Isohanni J Haapaniemi J . Electric field navigated transcranial magnetic stimulation for chronic tinnitus: a pilot study. Int J Audiol. 2015;54:899–909. 26086944 10.3109/14992027.2015.1054041

[29] Lamusuo S Hirvonen J Lindholm P . Neurotransmitters behind pain relief with transcranial magnetic stimulation—PET evidence for release of endogenous opioids. Eur J Pain. 2017;21:1505–1515. 28493519 10.1002/ejp.1052

[30] Lindholm P Lamusuo S Taiminen T . Right somatosensory cortex—a promising novel target for the treatment of drug-resistant neuropathic orofacial pain with repetitive transcranial magnetic stimulation. Pain. 2015;156:1276–1283. 25830924 10.1097/j.pain.0000000000000175

[31] Mylius V Ayache SS Ahdab R . Definition of DLPFC and M1 according to anatomical landmarks for navigated brain stimulation: inter-rater reliability, accuracy, and influence of gender and age. Neuroimage. 2013;78:224–232. 23567888 10.1016/j.neuroimage.2013.03.061

[32] Teng S Guo Z Peng H . High-frequency repetitive transcranial magnetic stimulation over the left DLPFC for major depression: session-dependent efficacy: a meta-analysis. Eur Psychiatry. 2017;41:75–84. 28049085 10.1016/j.eurpsy.2016.11.002

[33] Dalton B Bartholdy S McClelland J . Randomized controlled feasibility trial of real versus sham repetitive transcranial magnetic stimulation treatment in adults with severe and enduring anorexia nervosa: the TIARA study. BMJ Open. 2018;8:e021531. doi:10.1136/bmjopen-2018-021531. PMC6082449 30012789

[34] Jassova K Albrecht J Papežova H . Repetitive transcranial magnetic stimulation (rTMS) treatment of depression and anxiety in a patient with anorexia nervosa. Med Sci Monit. 2018;24:5279–5281. 30057403 10.12659/MSM.908250 PMC6080581

[35] Thompson L . Treating major depression and comorbid disorders with transcranial magnetic stimulation. J Affect Disord. 2020;276:453–460. 32871677 10.1016/j.jad.2020.07.025 PMC7505211

[36] De Ridder D De Mulder G Menovsky T . Electrical stimulation of auditory and somatosensory cortices for treatment of tinnitus and pain. Prog Brain Res. 2007;166:377–388. 17956802 10.1016/S0079-6123(07)66036-1

[37] Martin DM McClintock SM Forster J . Does therapeutic repetitive transcranial magnetic stimulation cause cognitive enhancing effects in patients with neuropsychiatric conditions? A systematic review and meta-analysis of randomized controlled trials. Neuropsychol Rev. 2016;26:295–309. 27632386 10.1007/s11065-016-9325-1 PMC5949868

[38] Baruch N Burgess J Pillai M . Treatment for depression comorbid with dementia. Evid Based Ment Health. 2019;22:167–171. 31558560 10.1136/ebmental-2019-300113 PMC10231626

[39] Ahdab R Ayache SS Brugières P . Comparison of “standard” and “navigated” procedures of TMS coil positioning over motor, premotor and prefrontal targets in patients with chronic pain and depression. Neurophysiol Clin. 2010;40:27–36. 20230933 10.1016/j.neucli.2010.01.001

[40] Fitzgerald PB Hoy K McQueen S . A randomized trial of rTMS targeted with MRI based neuro-navigation in treatment-resistant depression. Neuropsychopharmacology. 2009;34:1255–1262. 19145228 10.1038/npp.2008.233

[41] Kobayashi M Fujimaki T Mihara B . Repetitive transcranial magnetic stimulation once a week induces sustainable long-term relief of central poststroke pain. Neuromodulation. 2015;18:249–254. 25906811 10.1111/ner.12301

[42] Kedzior KK Azorina V Reitz S . More female patients and fewer stimuli per session are associated with the short-term antidepressant properties of repetitive transcranial magnetic stimulation (rTMS): a meta-analysis of 54 sham-controlled studies published between 1997–2013. Neuropsychiatr Dis Treat. 2014;10:727–756. 24855360 10.2147/NDT.S58405 PMC4019615

